# Early lung ultrasound score combined with umbilical cord-blood procalcitonin improves 1-year prognostic stratification in preterm neonates with respiratory distress syndrome

**DOI:** 10.3389/fped.2026.1765207

**Published:** 2026-02-11

**Authors:** Lei Zheng, Hongyan Jing, Lihong Liu, Yang Gao, Lianyi Wang

**Affiliations:** 1 Department of Ultrasonography, First Hospital of Tsinghua University, Beijing, China; 2 Operating Room, First Hospital of Tsinghua University, Beijing, China

**Keywords:** lung ultrasound (LUS) score, preterm (birth), procalcitonin (PCT), prognosis, respiratory distress syndrome

## Abstract

**Background:**

Respiratory distress syndrome (RDS) remains a major cause of morbidity in very preterm infants. Lung ultrasound score (LUS) provides a bedside assessment of lung aeration and has demonstrated utility for early respiratory decision-making, but its prognostic performance for long-term outcomes is only moderate. Procalcitonin (PCT) measured in umbilical cord blood may reflect perinatal inflammatory exposure and risk of infection-related complications.

**Methods:**

We conducted a single-center prospective cohort study enrolling infants born at 24 + 0–33 + 6 weeks' gestation who were admitted to the NICU within 6 h of birth and were clinically diagnosed with RDS. Within 6 h after delivery, a standardized 12-zone LUS and umbilical cord-blood PCT were obtained. The primary endpoint was a composite of bronchopulmonary dysplasia, severe intraventricular hemorrhage, necrotizing enterocolitis, culture-proven sepsis occurring after 72 h of age, or all-cause death within 12 months' corrected age. Discrimination was evaluated using ROC analysis and DeLong tests. Time-to-first-event associations were examined using multivariable Cox regression. Internal validation used bootstrap optimism correction.

**Results:**

Among 290 infants, 110 (37.9%) reached the composite endpoint (event-free proportion 62.1%). LUS alone achieved an AUC of 0.76 (95% CI 0.70–0.82), and PCT alone an AUC of 0.78 (0.72–0.84). A logistic model combining LUS and log-transformed PCT improved discrimination to an AUC of 0.87 (0.83–0.92), outperforming each single marker (paired DeLong *p* < 0.001). At the Youden-optimal operating point, sensitivity was 82% and specificity 80%. In multivariable Cox analysis, the high-risk category defined by the combined model was independently associated with higher hazard of the composite outcome (HR 2.9, 95% CI 2.0–4.1), alongside lower gestational age, lower birthweight, and early-onset infection. Bootstrap optimism-corrected AUC was 0.86.

**Conclusions:**

In preterm infants with RDS, early integration of 12-zone LUS and cord-blood PCT improves prediction of 12-month major morbidity or death compared with either marker alone. This bedside approach may support early risk stratification. External validation and impact studies are needed before score-guided management is recommended.

## Introduction

Respiratory distress syndrome (RDS) in preterm infants is primarily caused by a deficiency of pulmonary surfactant, which leads to atelectasis, intrapulmonary shunting, hypoxemia, and hypoventilation, ultimately contributing to conditions such as bronchopulmonary dysplasia (BPD) and pulmonary hypertension (1, 2). RDS occurs in approximately 30% of infants born before 28 weeks of gestational age (GA) (3). This condition contributes to 20%–40% early mortality and is a precursor to chronic disabilities such as BPD and neurodevelopmental delays (4, 5). Despite advancements in neonatal care, current prognostic tools for RDS offer limited predictive power with an area under the curve (AUC) of approximately 0.65 (5). Early risk scores like CRIB II also lack specificity for pulmonary conditions (5). There is a pressing need for bedside, radiation-free, and operator-independent diagnostic tools to improve the prognostic accuracy for RDS in preterm infants (5). The incidence of RDS is inversely related to gestational age and birth weight, with a higher prevalence in infants born before 28 weeks and those with very low birth weights (3, 6). Factors such as maternal diabetes, cesarean delivery, and multiple births further increase the risk of RDS (3, 7). While antenatal corticosteroids and postnatal surfactant therapy have reduced the incidence and severity of RDS, it remains a leading cause of morbidity and mortality in preterm infants (2, 8). The development of new diagnostic and therapeutic strategies is crucial to further improve outcomes for these vulnerable infants (8).

The emergence of lung ultrasound scoring (LUS) in neonatal care has been pivotal in assessing RDS in preterm infants, offering a non-invasive, real-time method to quantify lung aeration and predict the need for surfactant therapy. The 6-zone method is simpler to evaluate lung aeration, while the 12-zone approach provides a more comprehensive assessment but is more time-consuming (9, 10). Meta-analyses have demonstrated that LUS is effective in predicting the need for surfactant administration (11). Umbilical cord-blood PCT rises early in the course of intrauterine inflammation and early-onset sepsis and can outperform CRP-based algorithms in some cohorts, although optimal thresholds vary with gestational age and timing of sampling (12). Elevated PCT has been linked to early-onset sepsis and to later respiratory or hemodynamic complications in very preterm infants (13, 14). These findings underscore the need for integrated diagnostic approaches combining LUS and biomarkers to enhance the management of neonatal respiratory conditions.

Structural and biochemical biomarkers interrogate distinct facets of neonatal respiratory disease, and their integration may therefore yield a more comprehensive early risk signal than either alone. We hypothesized that combining LUS with PCT measured within six hours of birth would sharpen prognostic accuracy by capturing both pathophysiological axes concurrently. Our primary aim is to determine whether the LUS + PCT model improves discrimination of the 12-month composite endpoint relative to either marker alone. Secondary objectives were to derive clinically usable operating points for early risk stratification, to evaluate each component outcome and resource-use outcomes, and to identify additional independent risk factors.

## Methods

### Study design and setting

We conducted a prospective cohort study in the First Hospital of Tsinghua University in China from March 2021 to March 2022. The study received approval from the ethics committee of the First Hospital of Tsinghua University and conformed to the principles of the Declaration of Helsinki. All data were de-identified before analysis, and access was restricted to authorized investigators. Written informed consent was obtained from a parent or legal guardian before any study procedures were undertaken.

### Eligibility criteria

Infants were eligible if they were born at 24 + 0–33 + 6 weeks' gestation, admitted to the NICU within 6 h of delivery, and met standard clinical and radiographic criteria for RDS as assessed by the attending neonatologist. RDS was defined by: (1) onset of respiratory distress (tachypnea, nasal flaring, chest retractions and/or grunting) within the first 6 h of life; (2) requirement for supplemental oxygen or respiratory support to maintain target saturations; and (3) a characteristic chest radiograph showing a diffuse reticulogranular “ground-glass” pattern with air bronchograms and reduced lung volumes. Infants with clinical and radiographic features more suggestive of transient tachypnea, meconium aspiration, primary pneumonia or other non-RDS diagnoses were not enrolled. We excluded infants with major congenital malformations (such as congenital diaphragmatic hernia, pulmonary hypoplasia or complex congenital heart disease), proven TORCH infection, chromosomal disorders or severe perinatal asphyxia defined by a five-minute Apgar score of three or less. Families who declined consent or whose infants lacked either the lung ultrasound or procalcitonin measurement were also excluded.

### Sample-size calculation

We prespecified a recruitment target to compare discriminatory performance (AUC) of the combined LUS plus PCT model against a single-marker approach. Assuming an area under the receiver-operating-characteristic curve (AUC) of 0.75 for the leading single marker and 0.85 for the combined model (13), with a two-sided alpha of 0.05% and 80% power, Hanley and McNeil's method indicated that at least 154 infants with and 154 without the composite endpoint were required. Allowing for a 10% attrition rate, we set a recruitment target of 343 infants. Because recruitment was limited by the prespecified study period, 290 eligible infants were ultimately enrolled, of whom 110 experienced the composite endpoint.

### Exposure measurements

*Lung ultrasound score (LUS)*: LUS was performed within 6 h of birth by credentialed sonographers blinded to PCT results and to follow-up outcome status (The 6 h window primarily reflects the timing needed to complete the bedside LUS after initial cardiorespiratory stabilization and NICU admission). Scanning used a GE LOGIQ S8 9.0-14.0Mhz linear transducer following a standardized 12-zone protocol (anterior, lateral, and posterior zones bilaterally). Each zone was scored 0–3 (0 = normal aeration; 1 = interstitial pattern; 2 = severe interstitial pattern/coalescent B-lines; 3 = consolidation), yielding a total score range 0–36 as previously described (15). Inter-rater reliability was assessed in a pilot set (quadratic-weighted κ = 0.83). The standardized 12-zone LUS score was calculated and recorded for research purposes and was not communicated to the treating clinical team in real time, and it did not influence bedside clinical decision-making.

*Umbilical cord-blood PCT*: Cord blood was collected immediately after clamping, prior to postnatal therapeutic interventions, centrifuged within 30 min, and PCT was measured using the BRAHMS PCT III chemiluminescent platform. Analytical range was 0.02–100 ng/mL within-run CV <6%. For modeling, PCT was log-transformed due to right-skewness. Cord-blood PCT testing was performed for the purposes of this research protocol, and results were not returned to the treating team in real time and therefore did not influence clinical management.

### Covariates

Maternal and neonatal baseline data, including gestational age, birth-weight, sex, mode of delivery, antenatal corticosteroid exposure, maternal intrapartum antibiotic exposure, suspected/clinical chorioamnionitis or maternal fever/infection, prolonged rupture of membranes, hypertensive disorders of pregnancy, gestational diabetes, surfactant administration, ventilation parameters and early-onset infection status, were extracted from the electronic medical record. Dual data entry with automated logic checks ensured accuracy, and discrepancies were resolved against source documents.

*Early-onset infection*: Early-onset infection was defined as clinician-diagnosed infection/sepsis within 72 h of birth based on institutional criteria (maternal risk factors and neonatal clinical signs), with or without culture confirmation, and antibiotic treatment consistent with NICU protocol.

### Outcomes

The primary outcome was a composite of bronchopulmonary dysplasia, assessed at 36 weeks' postmenstrual age using a severity-based definition aligned with contemporary NICHD/NRN criteria (16), intraventricular hemorrhage grade III or IV, necrotizing enterocolitis stage II or higher, culture-proven sepsis occurring after 72 h of age or all-cause death within 12 months corrected age. Secondary outcomes comprised each component of the composite, total days of hospitalization, duration of mechanical ventilation and re-hospitalization within the first year. Secondary analyses also included deriving clinically usable cut-off values for the combined model and examining additional independent risk factors.

### Statistical analysis

Normality of continuous variables was assessed using visual inspection and the Shapiro–Wilk test to determine whether results are summarized as mean ± SD or median (IQR), and categorical variables as frequencies with percentages. Group differences were assessed using Student's *t*-test or the Mann–Whitney *U*-test for continuous data and *χ*² or Fisher's exact test for categorical data. Missing values, which accounted for less than five per cent of any variable, were addressed with multiple imputation by chained equations (*m* = 20).

A logistic regression model combined LUS and log-transformed PCT to generate individual predicted risk probabilities. Discrimination was quantified by the AUC with 95% CI. AUCs were compared using paired DeLong tests. Multiple comparisons were controlled using the Benjamini–Hochberg false discovery rate (FDR). The Youden index was used to define a single operating point for sensitivity/specificity and to construct a binary “high-risk vs. low-risk” classification used for Kaplan–Meier displays and subgroup risk ratios.

*Time-to-event analysis*: Cox proportional hazards regression estimated associations with time to first composite event, adjusting for clinically relevant covariates. Proportional hazards assumptions were assessed using Schoenfeld residuals.

*Internal validation and sensitivity analyses*: Model performance was internally validated by 1,000 bootstrap resamples with optimism correction. Prespecified sensitivity analyses included using a 6-zone LUS scheme and restricting analyses to complete cases.

## Results

### Participant flow

**Figure 1:**
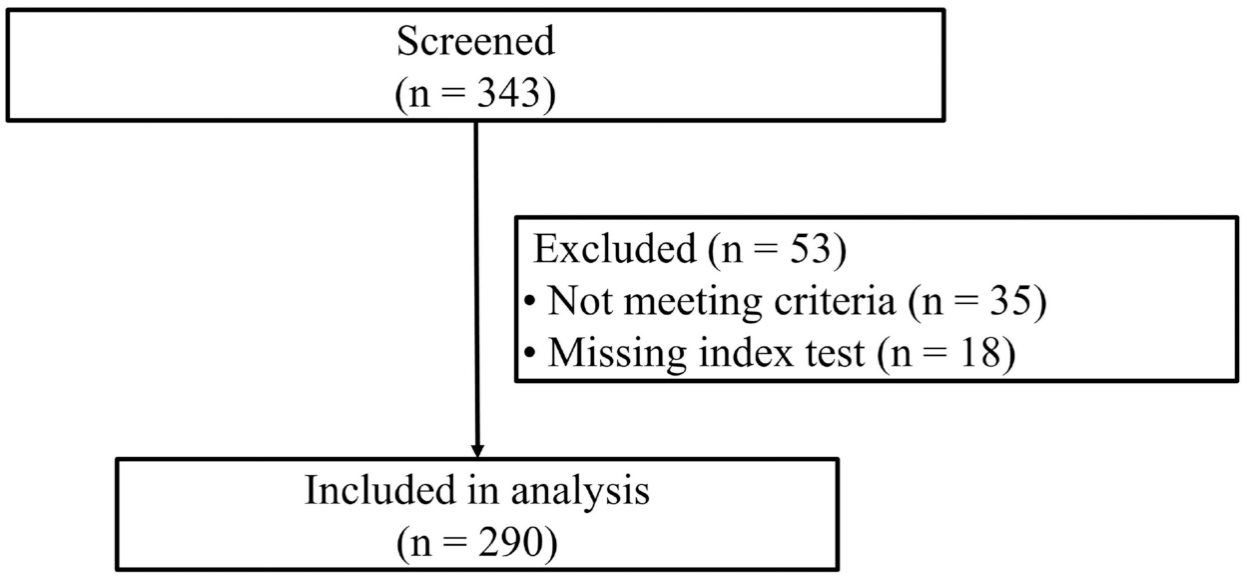
Study flow diagram of study recruitment and follow-up. A total of 343 preterm infants were screened for eligibility. Fifty-three were excluded—35 for not meeting inclusion criteria and 18 for missing an index test (either LUS or umbilical cord-blood PCT). This left left 290 infants in the final analytic cohort. All enrolled participants completed the 12-month follow-up with no losses or withdrawals.

A total of 343 preterm infants were screened. Fifty-three were excluded (35 not meeting eligibility criteria and 18 missing either LUS or PCT), leaving 290 (85.3%) infants with both index tests within 6 h for analysis (Figure 1). All included infants completed 12-month follow-up.

### Baseline characteristics

Infants reaching the composite endpoint (110/290, 37.9%) had lower gestational age (27.8 ± 2.5 vs. 29.5 ± 2.0 weeks, *p* = 0.001) and lower birthweight [1,125 [741–1,424] vs. 1,247 [873–1,548] g, *p* = 0.031] than those who remained event-free. The time to LUS examination was performed similarly after birth [median 2.2 (1.4–3.3) vs. 2.4 (1.6–3.5) hours, *p* = 0.23], while cord-blood PCT was collected at delivery. They more often had a 1 min Apgar score <7 (32.7% vs. 17.8%, *p* = 0.013). Repeat surfactant was less frequent in the event group (28.2% vs. 45.0%, *p* = 0.004) (Table 1). Maternal/perinatal baseline factors were also summarized in Table 1.

**Table 1:** Baseline characteristics.

Variable	No Event (*n* = 180)	Event (*n* = 110)	*p*
Gestational age (wk), mean ± SD	29.5 ± 2.0	27.8 ± 2.5	0.001
Birth-weight (g), median (IQR)	1,247 (873–1,548)	1,125 (741–1,424)	0.031
Gender			0.48
Male, *n* (%)	91 (50.6%)	61 (55.5%)	
Female, (*n* (%)	89 (49.4%)	49 (44.5%)	
Caesarean delivery, *n* (%)	117 (65.0%)	67 (60.9%)	0.57
Time-to-LUS (hours)			0.23
Mean ± SD	2.4 ± 1.3	2.6 ± 1.4	
Median (IQR)	2.2 (1.4–3.3)	2.4 (1.6–3.5)	
1 min Apgar <7, *n* (%)	32 (17.8%)	36 (32.7%)	0.013
Surfactant at birth, *n* (%)	171 (95.0%)	105 (95.5%)	0.87
Repeat surfactant, *n* (%)	81 (45.0%)	31 (28.2%)	0.004
Maternal/Perinatal factors			
Antenatal corticosteroids			0.78
None	15 (8.3%)	10 (9.1%)	
Partial	40 (22.2%)	28 (25.5%)	
Complete	125 (69.4%)	72 (65.5%)	
Maternal intrapartum antibiotic exposure			0.64
Yes	100 (55.6%)	65 (59.1%)	
No	80 (44.4%)	45 (40.9%)	
Suspected/clinical chorioamnionitis or maternal fever/infection	20 (11.1%)	15 (13.6%)	0.65
Prolonged rupture of membranes	35 (19.4%)	25 (22.7%)	0.60
Hypertensive disorders of pregnancy/preeclampsia			0.82
Yes	36 (20.0%)	20 (18.2%)	
No	144 (80.0%)	90 (81.8%)	
Gestational diabetes			0.86
Yes	14 (7.8%)	10 (9.1%)	
No	166 (92.2%)	100 (90.9%)	
Multiple gestation			0.66
Yes	48 (26.7%)	26 (23.6%)	
No	132 (73.3%)	84 (76.4%)	

### Primary outcome occurrence and timing

The composite endpoint occurred in 110 infants, the median time to first event was 21 days (IQR 9–35), and 72% of first events occurred during the index hospitalization. Overall, 180 infants (62.1%) remained free of the composite endpoint through 12 months' corrected age.

### Discrimination of LUS, PCT, and the combined model

**Figure 2:**
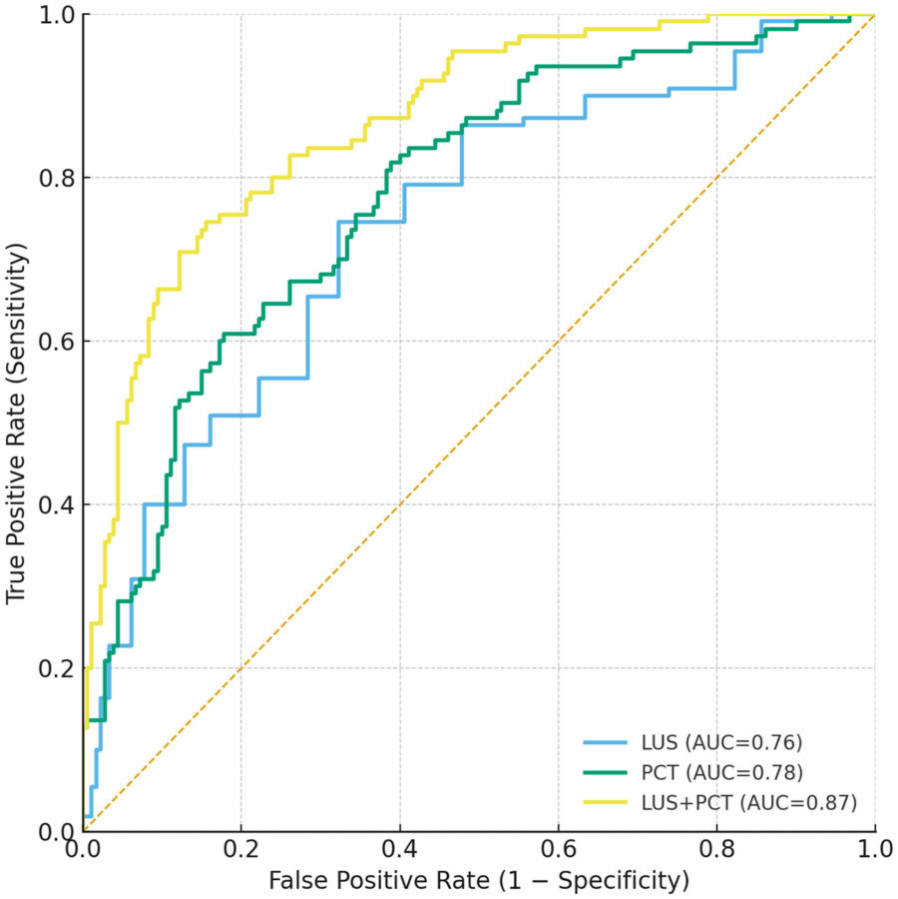
Receiver-operating-characteristic (ROC) curves for predicting the 12-month composite outcome. Curves compare the discriminative performance of LUS alone (AUC 0.76, 95% CI 0.70–0.82), PCT alone (AUC 0.78, 95% CI 0.72–0.84) and the combined LUS + PCT logistic model (AUC 0.87, 95% CI 0.83–0.92). The diagonal dashed line represents no-discrimination (AUC 0.50). The combined model significantly outperformed each single marker (paired DeLong tests, both *p* < 0.001, FDR-adjusted *q* < 0.005). AUC, area under the curve; CI, confidence interval; LUS, lung ultrasound score; PCT, procalcitonin.

Receiver-operating-characteristic analysis demonstrated AUCs of 0.76 (95% CI 0.70–0.82) for LUS alone and 0.78 (0.72–0.84) for PCT alone, the latter not significantly superior (DeLong *p* = 0.35, FDR-adjusted *q* = 0.350) (Figure 2 and Table 2). Combining LUS with log-transformed PCT in a logistic model increased the AUC to 0.87 (0.83–0.92), exceeding each single marker (both *p* < 0.001, *q* < 0.005). At the Youden-optimal threshold the combined model achieved 82% sensitivity, 80% specificity, a positive predictive value of 0.69 and a negative predictive value of 0.89, while calibration across risk deciles was excellent (Hosmer–Lemeshow *χ*² = 6.4, *p* = 0.60) (Table 2).

**Table 2:** Diagnostic performance for 12-month composite outcome.

Model	AUC (95% CI)	Sens	Spec	PPV	NPV	Youden *J*	DeLong *p* vs. LUS	DeLong *p* vs. PCT	FDR *q*
LUS	0.76 (0.70–0.82)	0.70	0.68	0.55	0.81	0.38	—	—	—
PCT	0.78 (0.72–0.84)	0.72	0.70	0.57	0.83	0.42	0.35	—	0.350
LUS + PCT	0.87 (0.83–0.92)	0.82	0.80	0.69	0.89	0.62	<0.001	<0.001	0.017

### Multivariable time-to-event associations

**Figure 3:**
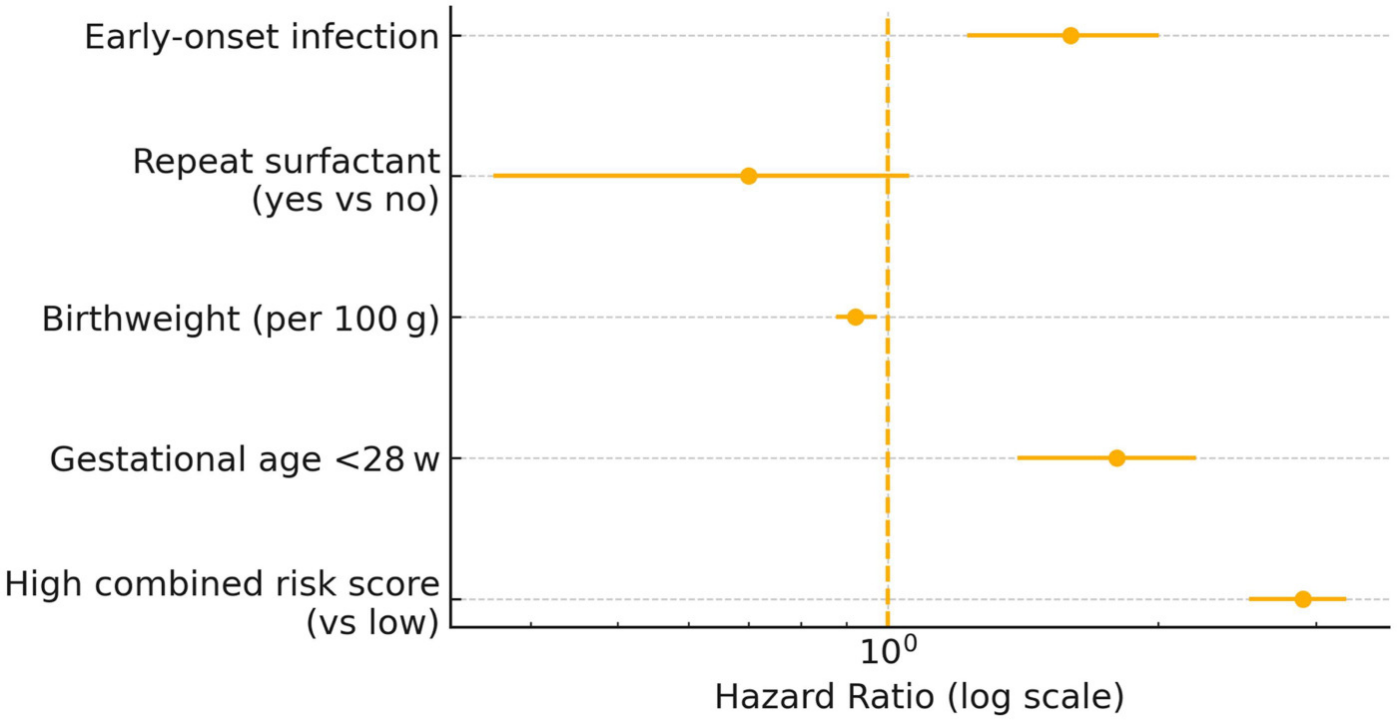
Adjusted hazard ratios for predictors of the 12-month composite outcome. Forest plot derived from the multivariable Cox proportional-hazards model shows that a high combined LUS + PCT risk score was the strongest independent predictor (HR 2.9, 95% CI 2.0–4.1), followed by gestational age <28 weeks (HR 1.8, 95% CI 1.2–2.7) and early-onset infection (HR 1.6, 95% CI 1.1–2.4). Higher birth-weight was protective (HR 0.92 per 100 g increase, 95% CI 0.88–0.97), whereas repeat surfactant therapy showed a borderline association with lower risk (HR 0.70, 95% CI 0.50–1.00). The vertical dotted line denotes the null value (HR 1.0); error bars represent 95% CIs plotted on a logarithmic scale. CI, confidence interval; HR, hazard ratio; LUS, lung ultrasound score; PCT, procalcitonin.

After adjustment for gestational age, birth-weight, repeat surfactant, and early-onset infection, a high combined risk score remained the strongest independent predictor of the composite endpoint [hazard ratio (HR) 2.9, 95% CI 2.0–4.1, *q* < 0.001]. Gestational age <28 weeks (HR 1.8, 95% CI 1.2–2.7, *q* = 0.009) and early-onset infection (HR 1.6, 95% CI 1.1–2.4, *q* = 0.025) were additional risk factors, whereas each 100-g increase in birth-weight reduced risk by 8 % (HR 0.92, 95 % CI 0.88–0.97). The proportional-hazards assumption was satisfied (global Schoenfeld *p* = 0.42) (Table 3 and Figure 3).

**Table 3:** Multivariable Cox regression for time to first adverse event.

Variable	HR	95% CI	*p*	FDR *q*
High combined risk score (vs. low)	2.9	2.0–4.1	<0.001	<0.001
Gestational age <28 wk	1.8	1.2–2.7	0.003	0.009
Birth-weight (per 100 g)	0.92	0.88–0.97	0.001	0.003
Repeat surfactant (no vs. yes)	0.70	0.50–1.00	0.054	0.072
Early-onset infection	1.6	1.1–2.4	0.015	0.025

### Component outcomes

Bronchopulmonary dysplasia was the most common individual complication (62/290 cases, 21.4%), followed by culture-proven sepsis (46/290 cases, 15.9%), severe intraventricular hemorrhage (grade ≥ III; 18/290, 6.2%), and necrotizing enterocolitis (stage ≥ II; 14/290, 4.8%). High-risk infants, as defined by the combined score, had significantly higher incidences of every component, with risk ratios ranging from 1.9 for severe intraventricular hemorrhage (95% CI 1.0–3.5, *q* = 0.060) to 2.8 for all-cause death (95% CI 1.5–5.2, *q* = 0.008); they also experienced median hospital stays and ventilation durations that were 12 days and 3 days longer, respectively (both *p* < 0.01) (Table 4).

**Table 4:** Incidence of individual components and risk ratios.

Outcome	Events, *n* (%)	Risk Ratio	95% CI	*p*	FDR *q*
BPD	62 (21.4%)	2.3	1.6–3.3	1 × 10^−4^	0.001
IVH ≥ III	18 (6.2%)	1.9	1.0–3.5	0.045	0.060
NEC ≥ II	14 (4.8%)	2.5	1.1–5.7	0.020	0.040
Sepsis	46 (15.9%)	2.0	1.4–2.9	0.003	0.008
All-cause death	19 (6.6%)	2.8	1.5–5.2	0.002	0.008

### Sensitivity analyses and safety

Results were robust when the 6-zone LUS scheme was substituted (combined AUC = 0.86), when only complete cases were analyzed (*n* = 277), and across gestational-age strata, with no significant interaction between risk score and gestational age (interaction *p* = 0.27). Bootstrap optimism-correction yielded an internally validated AUC of 0.86, confirming model stability.

No complications attributable to lung ultrasound scanning or cord-blood sampling were observed, and no protocol deviations occurred during the study period.

## Discussion

This prospective cohort study set out to determine whether early integration of a structural bedside marker (lung ultrasound score, LUS) with a biochemical marker of systemic inflammation (umbilical cord-blood procalcitonin, PCT) could enhance long-term risk stratification in pre-term infants with RDS. Among 290 eligible infants, 37.9% experienced the composite endpoint of major morbidity or death within 12 months. The combined LUS + PCT model achieved an area under the ROC curve of 0.87, with 82% sensitivity and 80% specificity at the optimal Youden threshold, substantially outperforming either biomarker alone. In adjusted Cox analysis, a high combined risk score independently conferred nearly a three-fold increase in hazard for the composite outcome. This investigation demonstrated that coupling lung aeration imaging with an early inflammatory signal can predict one-year morbidity and mortality in pre-term RDS, underscoring the clinical utility of a multimodal approach for early bedside decision-making in the NICU.

LUS offers an immediate, radiation-free quantification of pulmonary aeration that correlates with both the severity of neonatal RDS and later BPD  (17–22). PCT, in contrast, rises within hours of systemic infection and reliably signals neonatal sepsis and downstream complications such as necrotizing enterocolitis or death (13, 14, 23). By integrating these structural and biochemical signals within the first six hours after birth, our combined model captures two converging pathophysiological axes: impaired lung aeration and systemic inflammatory burden. This timing is clinically critical, because it precedes many decisive interventions—repeat surfactant dosing for refractory RDS or targeted antibiotics for suspected infection  (17). Experimental work further supports this dual-axis approach: hypo-ventilated lung regions increase bacterial translocation and amplify systemic cytokine release, an effect mitigated by lung-protective ventilation strategies  (24–26). Such synergy plausibly explains why our LUS + PCT model achieved a markedly higher AUC (0.87) and stronger independent hazard (HR 2.9) than either marker alone, delivering 82% sensitivity and 80% specificity at the bedside.

Our findings also advance the literature on prognostic tools for pre-term RDS. Prior studies report AUCs of 0.72–0.80 for LUS in predicting the need for mechanical ventilation or surfactant therapy  (25, 26), while biomarker-only approaches using PCT or C-reactive protein (CRP) seldom exceed an AUC of 0.80 for detecting sepsis, severe intraventricular hemorrhage or necrotizing enterocolitis (27). To date, neonatal studies have primarily evaluated LUS and inflammatory biomarkers separately. Outside the NICU, combined LUS + CRP strategies for respiratory infections are only beginning to be explored (28). By providing data that link early LUS + PCT measurements to a one-year composite of BPD, severe IVH, stage ≥ II NEC, culture-proven sepsis and death, our cohort fills this critical evidence gap  (29–32). The improved discrimination of our model in this cohort suggests potential utility for early beside risk stratification. However, external validation is required before clinical adoption.

Nonetheless, several caveats warrant consideration. The convenience sample was drawn from a single center in China, so generalizability to other ethnic or health-care settings may be limited. Residual confounding from unmeasured factors could persist despite multivariable adjustment; procalcitonin was measured at only one time point, precluding kinetic analyses; and the 12-month horizon does not capture later neuro-developmental sequelae. We did not reach the planned recruitment target and observed fewer composite events than anticipated in the initial AUC-based planning. As a result, the study may be underpowered to detect smaller differences in discrimination and the estimated cut-offs/performance metrics may have wider uncertainty. Cord-blood PCT may be influenced by maternal/perinatal factors (e.g., maternal infection/inflammation, intrapartum antibiotics, and delivery-related stressors). Although we report these baseline factors, residual confounding related to perinatal exposures cannot be fully excluded. Because LUS was obtained after initial stabilization, findings may vary with timing relative to early surfactant and ventilatory interventions. Clinically, a combined LUS + PCT score exceeding the optimal cut-off within six hours of birth could justify earlier repeat surfactant, intensified infection surveillance and more informed counselling of parents, all with minimal additional cost because both assessments rely on bedside ultrasound and routine cord-blood sampling; embedding the algorithm in NICU decision-support software or a smartphone calculator would further streamline implementation. Future work should externally validate the model in diverse high- and middle-income settings, explore machine-learning extensions incorporating cytokines such as IL-6 or sTREM-1, test score-guided management in randomized trials and extend follow-up to 24–36 months to determine whether early risk stratification translates into improved neuro-developmental outcomes.

Early integration of a 12-zone lung-ultrasound score with cord-blood procalcitonin, obtained within six hours of birth, markedly enhances prediction of 12-month morbidity and mortality in pre-term infants. This low-cost, bedside-ready approach is feasible for routine NICU workflow and provides actionable risk tiers that can trigger timely respiratory and antimicrobial interventions. Multicenter validation and interventional trials are now warranted to confirm clinical benefits and facilitate widespread adoption.

## Data Availability

The raw data supporting the conclusions of this article will be made available by the authors, without undue reservation.
